# Bronchial Responsiveness to Dry Air Hyperventilation in Smokers May Predict Decline in Airway Status Using Indirect Methods

**DOI:** 10.1007/s00408-012-9448-y

**Published:** 2013-01-25

**Authors:** Peter Blomstrand, Susanne Ekedahl, Birgitta Schmekel

**Affiliations:** 1 Department of Clinical Physiology, County Hospital Ryhov, Jönköping, 551 85 Sweden; 2 Rosenlund Primary Care Unit, Jönköping, 551 85 Sweden; 3 Division of Clinical Physiology, Department of Medicine and Health, Faculty of Health Sciences, Linköping University; and Department of Clinical Physiology, County Council of Östergötland, Linköping, Sweden

**Keywords:** Bronchial hyperresponsiveness, Impulse oscillometry, Isocapnic hyperventilation of dry air, Resonant frequency, Tobacco smoke

## Abstract

**Background:**

Disabling respiratory symptoms and rapid decline of lung function may occur in susceptible tobacco smokers. Bronchial hyperresponsiveness (BHR) elicited by direct challenge methods predicts worse lung function outcomes. The aim of this study was to evaluate whether BHR to isocapnic hyperventilation of dry air (IHDA) was associated with rapid deterioration in airway status and respiratory symptoms.

**Methods:**

One hundred twenty-eight smokers and 26 age- and sex-matched healthy individuals with no history of smoking were investigated. All subjects completed a questionnaire. Spirometry and impulse oscillometry (IOS) measurements were recorded before and after 4 min of IHDA. The tests were repeated after 3 years in 102 smokers and 11 controls.

**Results:**

Eighty-five smokers (66 %) responded to the challenge with a ≥2.4-Hz increase in resonant frequency (*F*_res_), the cutoff limit defining BHR, as recorded by IOS. They had higher *F*_res_ at baseline compared to nonresponding smokers [12.8 ± 3.2 vs. 11.5 ± 3.4 Hz (*p* < 0.05)] and lower FEV_1_ [83 ± 13 vs. 89 ± 13 % predicted (p < 0.05)]. Multivariable logistic regression analysis indicated that wheezing (odds ratio = 3.7, *p* < 0.01) and coughing (odds ratio = 8.1, *p* < 0.05) were significantly associated with hyperresponsiveness. An increase in *F*_res_ was recorded after 3 years in responding smokers but not in nonresponders or controls. The difference remained when subjects with COPD were excluded.

**Conclusions:**

The proportion of hyperresponsive smokers was unexpectedly high and there was a close association between wheezing and coughing and BHR. Only BHR could discriminate smokers with rapid deterioration of airway status from others.

## Introduction

Tobacco smoking may result in significant limitation of physical performance in susceptible subjects. Hindering symptoms such as dyspnea, coughing, and attacks of wheezing and airway obstruction may occur, and some smokers may experience a faster-than-normal decline of lung function and ultimately chronic obstructive pulmonary disease (COPD), a disorder characterized by systemic and airway mucosal inflammation [1–3]. Although bronchial hyperresponsiveness (BHR) may occur in many diseases [4], it is most common in patients with inflammatory disorders of the airway mucosa. It has been reported in smokers with COPD that subjects with a faster-than-normal decline of lung function may be identified by hyperresponsiveness to methacholine or histamine [5, 6]. BHR is also an independent predictor of mortality in patients with COPD [7]. An extensive search for surrogate markers capable of predicting outcomes in terms of lung function in smokers has been conducted, and numerous studies on serum or sputum markers of inflammation, as well as on various tests of BHR, have been published. BHR, usually evaluated by inhalation of methacholine or histamine, has been found to occur in up to 85 % of smokers with COPD [5, 8, 9]. In contrast, BHR elicited by indirect challenge methods, such as isocapnic hyperventilation of cold or dry air (IHDA), was reported to occur much less frequently; only 16 % of smokers were reported to respond to IHDA with increased airway tonus [10, 11].

Responses to bronchial challenges have been measured mostly in terms of forced expiratory volume in 1 second (FEV_1_), a technique that requires considerable patient cooperation. A deep inhalation preceding and/or a forced expiration during a FEV_1_ maneuver may alter bronchial tone for time periods up to 6 min; this may reduce the ability of tests to correctly identify BHR [12, 13]. Impulse oscillometry (IOS) is an alternative technique to measure airway status that requires minimal patient cooperation. IOS is particularly suitable for serial measurements such as those required during a bronchial challenge [14, 15]. Airway resistance, reactance, and resonant frequency (*F*_res_) are the outcome data; values of *F*_res_ are closely related to those of airway resistance and are defined by the frequency at which inertial forces are equal and opposite to elastic forces (i.e., reactance is zero).

The aim of this study was to evaluate whether BHR to IHDA, as measured by means of IOS, was associated with deterioration in airway status relative to baseline in a 3-year follow-up study. We also examined whether the presence of BHR was associated with subjectively perceived respiratory symptoms or reduced pulmonary function in smokers.

## Materials and Methods

### Subjects

One hundred ninety-eight smokers attending 11 general practitioners’ offices in Jönköping County, Sweden, were invited to participate in the study. The inclusion criterion was regular, daily tobacco smoking over at least 25 years. Subjects with severe cardiovascular, pulmonary (other than COPD), or systemic disease, those treated with corticosteroids (budesonid ≥800 μg or the equivalent), and those with physician-diagnosed asthma were not included. Forty-four subjects (30 women and 14 men) refrained from participation due to personal reasons. Data from a further 26 smokers (23 women and 3 men) were excluded because it was missing or the pulmonary function recordings were nonreproducible. A complete dataset was recorded for 76 women and 52 men (Table 1). Thirty-five age- and sex-matched subjectively healthy individuals with no history of smoking were enrolled as controls, nine of whom were excluded due to nonreproducible pulmonary function recordings. A complete dataset was recorded in 17 female and 9 male healthy controls. Baseline pulmonary function tests were repeated after approximately 3 years in 102 smokers and 11 healthy nonsmokers. Fifteen smokers had stopped smoking between 1 and 26 weeks prior to the first visit and 12 had ceased smoking >12 months prior to the second visit. Only four smokers refrained completely from smoking during the 3-year observation period. Body mass index (BMI) was defined as the subject’s body mass (kg) divided by the square of his or her height (m).

**Table 1:** Characteristics, respiratory symptoms, and pulmonary function in smokers and healthy volunteers with no smoking history

		Smokers (*n* = 128)	*p*	Healthy volunteers (*n* = 26)
Characteristics	Age (years)	58 ± 7	ns	57 ± 7
	Sex (female/male)	76/52	ns	17/9
	BMI	26 ± 4	***	23 ± 2
	β_2_-agonists	15	ns	0
Symptoms	Cough affected by weather	20	ns	2
	Phlegm cough without cold	71	**	5
	Phlegm cough in the morning	56	**	3
	Wheeze	86	***	2
	Breathing problems	32	**	0
	Shortness of breath on cold	54	***	1
Pulmonary function	FEV_1_ (% predicted)	85 ± 13	***	101 ± 7
	FEV_1_/VC (% predicted)	94 ± 11	***	103 ± 6
	*F*_res_ baseline (Hz)	12.4 ± 3.3	***	9.6 ± 1.3
	∆F_res_ (Hz)	4.1 ± 3.4	***	1.0 ± 1.0

Values are mean ± standard deviation or number of subjects, unless otherwise stated

*BMI* body mass index* β*_*2*_ *-agonists* subjects undergoing β_2_-agonist treatment, *FEV*_*1*_ forced expiratory volume during 1 second, *VC* vital capacity, *F*_res_ resonant frequency, ∆*F*_res_ change in *F*_res_ after bronchial challenge

*p < 0.05, **p < 0.01, ***p < 0.001, *ns* not statistically significant

All subjects completed a questionnaire regarding their medical history, including drug therapy, smoking habits, and respiratory symptoms. Three standardized questions concerning symptoms associated with COPD were selected from the International Primary Care Airways Group Handbook [16]: (1) Does the weather affect your cough? (2) Do you ever cough up phlegm from your chest when you do not have a cold? (3) Do you usually cough up phlegm from your chest in the morning? Three additional questions were asked: Do you usually experience wheezing? Do you usually have breathing problems? Do you usually experience shortness of breath when you have a cold?

A 3-week period free from the common cold was required to precede the tests. Patients were asked not to use β_2_-agonists, drink xanthine-containing beverages, or smoke during the 12 hours prior to the tests. All measurements were carried out during a single day at the Department of Clinical Physiology, County Hospital Ryhov, Jönköping, Sweden. The study complied with the Declaration of Helsinki and was approved by the Regional Ethics Research Committee at Linköping. All subjects gave written informed consent before inclusion in the study.

### Pulmonary Function Tests

Spirometry and IOS measurements were performed using a Masterscreen-IOS device (E. Jaeger GmbH, Wurzburg, Germany). FEV_1_ and vital capacity measurements were performed according to clinical routine and guidelines of the American Thoracic Society and European Respiratory Society [17, 18]. Spirometry data are given as a percentage of reference values as documented in a national nonsmoking reference population [19, 20]. The *F*_res_ was determined by means of IOS [14, 15, 21]. The subjects wore a nose clip and supported their cheeks with their hands. Pressure impulses were then applied to the respiratory system via a mouthpiece during tidal breathing. The responding signal was measured with a pneumotachograph and pressure transducer. *F*_res_ was calculated from the pressure-flow relationship by using a fast Fourier transformation. Measurements were repeated at least three times at baseline and a representative *F*_res_ value was selected. Measurements were also repeated 2, 4, and 6 min after IHDA, and the highest, most appropriate values were selected. Based on duplicate measurements of responses to challenge in healthy nonsmoking controls, a significant change in *F*_res_ and a cutoff limit for BHR were defined by an increase in *F*_res_ of ≥2.4 Hz (i.e., 3 × standard deviation [SD]_diff_), which corresponds to an increase of approximately 20 %.

### Hyperventilation Challenge

All subjects were encouraged to breath through a mouthpiece at a rate of ~28 breaths/min for 4 min. A gas mixture containing 21 % O_2_, 5 % CO_2_, and 74 % N_2_ was delivered through a calibrated rotameter to a meteorological reservoir balloon (Ailos Asthma Test 22 000; Karlstad, Sweden) and directed to the subject. The target ventilation was set to 24 × FEV_1_ (i.e., 70 % of maximal voluntary ventilation) and subjects were encouraged to deflate the balloon.

### Statistical Analysis

Data are presented as mean and standard deviation, 95 % confidence interval, or median [lower—upper quartile]. The Mann–Whitney *U* test was used to test differences among numerical variables, and Fisher’s exact test was used for binary variables. Univariable and multivariable logistic regression analyses were used to determine whether any variable was associated with BHR, and the results were expressed as odds ratios. Pearson’s correlation coefficient was calculated to study the association between responses to challenge and pulmonary function. Receiver-operated curve (ROC) analyses were performed to identify smokers with rapid deterioration in airway status. A *p*-value of ≤0.05 was considered to be statistically significant. Two-sided tests were used throughout. Statistical analyses were performed using the commercially available statistical programs STATISTICA version 9 (StatSoft Inc., Tulsa, OK, USA, www.statsoft.com) and SAS/Stat software version 9.2 of the SAS System for Windows (SAS Institute Inc., Cary, NC, USA). ROC analyses were performed using MedCalc Statistical Software (Mariakerke, Belgium).

## Results

Demographic characteristics, respiratory symptoms, and the results of pulmonary function tests in 128 smokers and 26 healthy nonsmoking volunteers are given in Table 1. All subjects were Caucasians. The smokers smoked 14 ± 8 cigarettes per day and had been smoking for 41 ± 7 years. Thirty-eight smokers smoked less than 10 cigarettes per day.

**Fig. 1:**
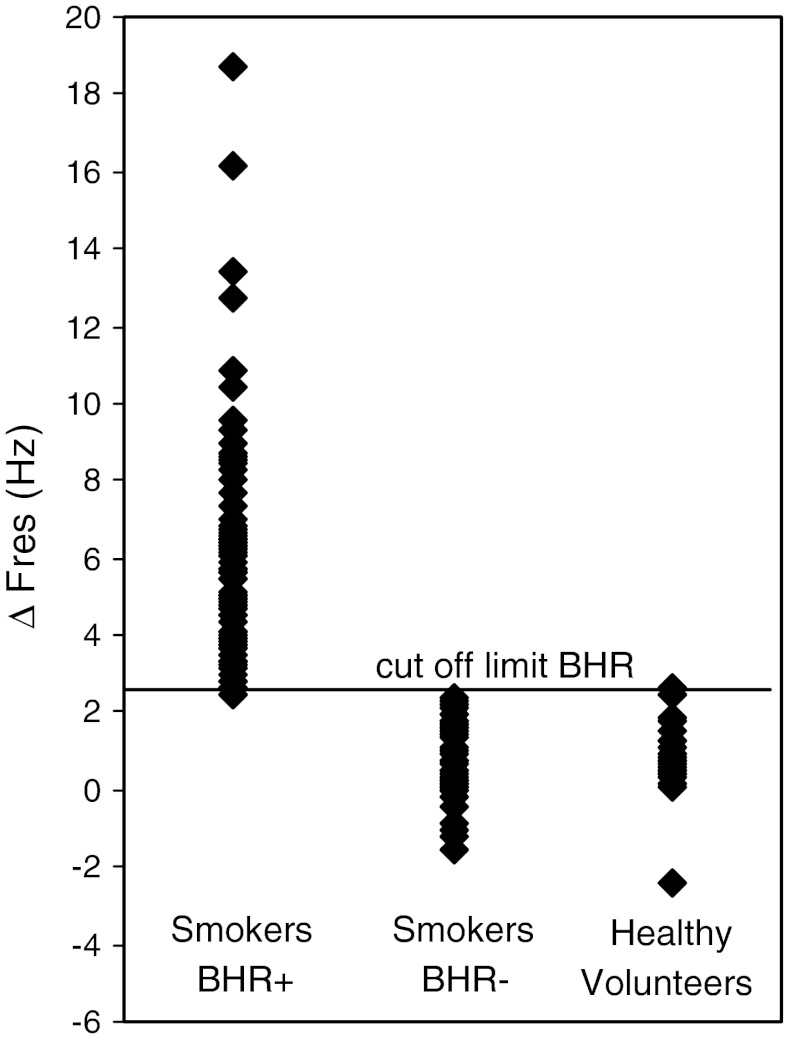
Scatterplot of change in resonant frequency (Δ*F*res) after dry air challenge in responding smokers (BHR+), nonresponding smokers (BHR−), and healthy volunteers with no smoking history

Eighty-five smokers showed significant increases in *F*_res_ (i.e., ≥2.4 Hz) after IHDA and were classified as “responders” (BHR+). The remaining 43 smokers constituted a group of “nonresponders” (BHR−; Fig. 1). Respiratory symptoms were more common in responders than in nonresponders, and responders also had worse pulmonary function at baseline than did nonresponders (*p* < 0.05; Table 2). Responses to challenge were not associated with baseline values of either *F*_res_ or FEV_1_ (*r* = 0.07, *p* > 0.05 or *r* = −0.13, *p* > 0.05). Female smokers outnumbered male smokers, and they had been regular smokers for a shorter period of time than male smokers (40 ± 7 vs. 43 ± 6 years, *p* < 0.05); however, there were no gender-related differences in pulmonary function or IHDA response. Twenty-nine smokers fulfilled the Global Initiative for Chronic Obstructive Lung Disease (GOLD) criteria for COPD, 24 of whom were responders [22].

**Table 2:** Characteristics, respiratory symptoms, and pulmonary function in smokers with (BHR+) and without (BHR−) bronchial hyperresponsiveness

		Smokers BHR+ (*n* = 85)	*p*	Smokers BHR− (*n* = 43)
Characteristics	Age (years)	57 ± 7	ns	58 ± 8
	Sex (female/male)	50/35	ns	26/17
	BMI	26 ± 4	ns	25 ± 3
	Smoke years	41 ± 7	ns	41 ± 7
	Cigarettes/day	14 ± 9	ns	13 ± 8
	β_2_-agonists	15	**	0
Symptoms	Cough affected by weather	19	**	1
	Phlegm cough without cold	54	**	17
	Phlegm cough in the morning	40	ns	16
	Wheeze	67	***	19
	Breathing problems	26	*	6
	Shortness of breath on cold	41	ns	13
Pulmonary function	FEV_1_ (% predicted)	83 ± 13	*	89 ± 13
	FEV_1_/VC (% predicted)	92 ± 11	*	98 ± 8
	*F*_res_ baseline (Hz)	12.8 ± 3.2	*	11.5 ± 3.4

Values are mean ± standard deviation or number of subjects, unless otherwise stated

*BMI* body mass index *β*_*2*_ *-agonists* subjects undergoing β_2_-agonist treatment, *FEV*_*1*_ forced expiratory volume during 1 second, *VC* vital capacity, *F*_res_ resonant frequency

*p < 0.05, **p < 0.01, ***p < 0.001, *ns* not statistically significant

Wheezing was the most commonly perceived symptom (Table 3), and wheezers had a higher body mass index (BMI), worse pulmonary function, and more pronounced responses to challenge than nonwheezers. Furthermore, coughing was associated with worse hyperresponsiveness (*p* < 0.001). In contrast, there was no association between coughing and BMI, longer tobacco smoke exposure, or current smoking habits.

**Table 3:** Characteristics and pulmonary function in smokers with and without subjectively perceived wheezing

		Wheezers (*n* = 86)	*p*	Nonwheezers (*n* = 42)
Characteristics	Age (years)	57 ± 8	ns	58 ± 7
	BMI	26 ± 4	**	24 ± 4
	Smoke years	41 ± 7	ns	41 ± 7
	Cigarettes/day	16 ± 8	***	10 ± 8
	β_2_-agonists	15	***	0
Pulmonary function	FEV_1_ (% predicted)	83 ± 14	**	90 ± 11
	FEV_1_/VC (% predicted)	93 ± 11	ns	96 ± 8
	*F*_res_ baseline (Hz)	12.9 ± 3.3	**	11.2 ± 3.0
	∆*F*_res_ (Hz)	4.9 ± 3.5	***	2.5 ± 2.6

Values are mean ± standard deviation or number of subjects, unless otherwise stated

*BMI* body mass index *β*_*2*_ *-agonists* subjects undergoing β_2_-agonist treatment, *FEV*_*1*_ forced expiratory volume during 1 second, *VC* vital capacity, *F*_res_ resonant frequency, ∆*F*_res_ change in *F*_res_ after bronchial challenge

*p < 0.05, **p < 0.01, ***p < 0.001, *ns* not statistically significant

A univariable logistic regression analysis was performed to analyze the association between BHR and respiratory symptoms, pulmonary function, and gender in smokers (Table 4). These variables were also used in a multivariable logistic regression analysis, the results of which indicated that wheezing (odds ratio = 3.7, *p* < 0.01) and coughing affected by weather (odds ratio = 8.1, *p* < 0.05) should remain in the logistic regression model; these factors were significantly associated with hyperresponsiveness. The odds ratio of BHR in smokers with a combination of symptoms of wheezing and weather-induced cough was 21.2 times that calculated in smokers not experiencing these symptoms.

**Table 4:** Odds ratios (OR) of bronchial hyperresponsiveness to hyperventilation of dry air associated with respiratory symptoms, pulmonary function, or gender presented with 95 % confidence intervals (95 % CI), based on univariable logistic regression in smokers

	OR	95 % CI	*p*
Cough affected by weather	12.3	[1.6–95.0]	0.01
Phlegm cough without cold	2.8	[1.3–5.9]	0.01
Phlegm cough in the morning	1.5	[0.7–3.2]	ns
Wheeze	4.7	[2.1–10.4]	0.0001
Breathing problems	2.7	[1.0–7.2]	0.05
Shortness of breath on cold	2.1	[1.0–4.7]	0.06
FEV_1_ < 80 % predicted	2.0	[0.9–4.6]	0.09
FEV_1_/VC < 70 %	1.9	[0.9–4.4]	0.09
Sex, female gender	0.9	[0.4–2.0]	ns

*FEV*_*1*_ forced expiratory volume during 1 second, *VC* vital capacity *ns* not statistically significant

One hundred two of the 128 smokers and 11 of the 26 healthy volunteers repeated the tests approximately 3 years after the first visit. Baseline pulmonary function, as determined by the pretest *F*_res_ value, deteriorated significantly more in responders [(median (lower to upper quartile, *p* = 0.03) = 1.1 (−0.6 to +4.0) Hz)] than in nonresponders [0.2 (−1.0 to +1.5) Hz] during this time period. The corresponding change in healthy volunteers was [−0.1 (−0.7 to +0.6) Hz]. There was no significant decline in FEV_1_ (% predicted) after 3 years in responding smokers, nonresponding smokers, or healthy volunteers [−1.1 (−6.0 to +4.0) % and −1.0 (−6.4 to +3.0) % vs. 1.5 (−7.6 to +5.2) %]. Responsiveness to IHDA, but none of the other tests or demographic data recorded at baseline during the first visit, was capable of discriminating between those who experienced worsening airway status and those who did not, as defined by *F*_res_ at baseline, after the 3-year observation period. There was a small but not statistically significant difference in deterioration of pulmonary function after 3 years between responding and nonresponding smokers when subjects with COPD were excluded from evaluation [0.7 (−0.6 to +3.9) vs. −0.1 (−0.9 to +1.6) Hz (*p* = 0.1)].

Based on the assumption that pulmonary function may deteriorate in some of the smokers, even during such a short period of time as 3 years, a ROC analysis was performed using significant worsening of the pretest value of *F*_res_ (i.e., increases in baseline *F*_res_ ≥ 2.4 Hz) over time as the classification variable. ROC analyses revealed an alternative cutoff level of ≥3.8 Hz for the *F*_res_ increase. Using the alternative cutoff level, 65 smokers instead of 85 would be defined as hyperresponsive to IHDA at the first visit. The majority (51 %) of all responding smokers had an *F*_res_ increase after challenge exceeding 3.8 Hz. This higher threshold level yielded an area under the ROC curve (AUC) of 0.66 (standard error [SE] = 0.05), sensitivity of 53 %, and specificity of 85 %. As a comparison, the corresponding AUC calculated using the main criterion (i.e., response to IHDA in individuals with *F*_res_ ≥ 2.4 Hz) was 0.63 (SE = 0.06), and the sensitivity and specificity were 47 and 92 %, respectively. Hyperresponsive smokers, as defined by the higher threshold level, experienced significantly greater worsening of the baseline airway status than those with *F*_res_ increases below this threshold (baseline *F*_res_ changes between the two study days [2.5 (−0.3 to +4.5) vs. 0.1 (−1.1 to +1.6) Hz (*p* = 0.005)]. The difference between the groups remained when smokers with COPD were excluded from evaluation [1.0 (−0.4 to +4.1) vs. 0.1 (−1.0 to +1.6) Hz (*p* = 0. 05)]. There were no differences in the baseline FEV_1_ values recorded during the second visit between these two subgroups of smokers (*p* > 0.05).

## Discussion

Thresholds for classification of results from bronchial challenge are arbitrary, and optimum cutoff levels must be chosen depending on the challenge method used [23]. Measurements of IHDA responses most often have been achieved by means of forced expirations, and a 12 % decrease in FEV_1_ has been reported to be an optimal cutoff level [23]. We used IOS for the detection of responses and defined a positive response as an increase in *F*_res_ of at least 2.4 Hz, corresponding to a 20 % increase. Eighty-five of 128 (66 %) smokers were then recognized as responders, and this threshold distinctly discriminated healthy individuals with no smoking history from responding smokers. Pulmonary function at baseline, as determined by *F*_res_, deteriorated significantly during a 3-year observation period in BHR+ smokers but not in BHR− smokers or healthy volunteers. Although increases in baseline *F*_res_ were modest, this still suggests that the cutoff limit of 2.4 Hz is clinically relevant, provided that a true deterioration of pulmonary function occurred during this relatively short period of time in BHR+ smokers, despite that FEV_1_ recordings were not significantly decreased in the follow-up visit. The fact that *F*_res_ increased significantly in BHR+ smokers during the observation period but FEV_1_ remained unchanged tends to confirm that the IOS technique is more sensitive than forced expirations in measuring changes in airway function [14, 21, 24]. Furthermore, as an additional sign of the higher sensitivity of the IOS method compared to FEV_1_, we found only a weak association between the levels of BHR and impaired pulmonary function at baseline, confirming the view that IOS recordings are independent of airflow and/or the prechallenge caliber of airways. A supplementary ROC analysis revealed that a *F*_res_ cutoff value of 3.8 Hz identified 65 of 128 smokers (51 %) as responders. The requirement of a smaller decrease in *F*_res_ to define BHR+ would capture more cases of reactive airways (increased sensitivity) but would also include some “normal” responses (decreased specificity). We found no major differences in accuracy calculated with these two different threshold levels, and therefore the final choice of cutoff level may remain unclear until a longer observation period has passed to enable confirmation of the clinical significance of the decline in airway status. Pulmonary function deteriorated in hyperresponsive smokers, even when subjects with COPD were excluded. Hyperresponsiveness to IHDA determined by IOS therefore appears to be a risk factor for deterioration of pulmonary function in smokers independent of baseline airway status or COPD.

The proportion of responders was unexpectedly high considering that most of our smokers had only mild signs of smoke-elicited injuries. The prevalence of BHR after hyperventilation of cold air was previously reported to be approximately 15 % of smokers with chronic bronchitis or COPD. However, responses to challenge were measured by means of FEV_1_, which may underestimate the true response [25]; this could explain the low proportion of responders among smokers upon indirect challenge in previous studies [10, 26, 27]. Therefore, the large variability in BHR prevalence might depend at least partly on the methodological limitations of the forced expiration technique, since airway tonus may change after a deep inspiration that precedes the FEV_1_ maneuver [28, 29]. Furthermore, responses to deep inspirations have been reported to decrease with COPD severity [30], and this may result in a larger variability in BHR prevalence depending on differences in age distribution among the various study populations. Therefore, it is concluded that variability in BHR prevalence among smokers may originate from differences in challenge methods, choice of threshold levels, and/or measurement techniques, apart from the phenotypical differences in populations of smokers.

There are methodological shortcomings of the IHDA technique per se that result from technical difficulties in measuring and controlling airflow or alternating volumes of exhaled warm humid air and inhaled dry cold air; this makes the determination of “challenge doses” complex. Although subjects were encouraged to reach the target level of ventilation, some of them failed and inhaled less than intended; this may have introduced a type II error leading to underestimation of the number of hyperresponsive subjects. Furthermore, the selection of subjects in our study was not population-based. We recruited smokers seeking primary health care, and selection bias theoretically might have occurred because patients with asthma or other diseases were excluded based mainly on clinical grounds. All our subjects were Caucasians and there might be racial differences in vulnerability to tobacco smoke [31]. We did not assess diet or passive tobacco smoke exposure during childhood, factors that may influence susceptibility. Twenty-three smokers tried to quit smoking but only four succeeded to refrain completely from smoking during the 3-year observation period. We could not show that temporary or permanent cessation of smoking changed the pulmonary function results between the two study days, probably because there were too few who refrained completely from smoking. Smoking cessation prevents accelerated decline in lung function in all smokers [32]. We did not exclude smokers who refrained from smoking. We also judged that the dropout rate of 20 % among our smokers in the 3-year observation period was fairly large (though acceptable). Despite the fairly large dropout rate, the number of remaining subjects allowed us to also use data recorded during the second visit to the laboratory.

Intermittent wheezing was the most common symptom reported by the smokers, and there was a close association between respiratory symptoms and BHR. Odds ratios for the presence of BHR ranged from 3 to 20 if there was a simultaneous history of either wheezing and/or coughing. This may suggest parallel events and/or common underlying mechanisms of these particular symptoms and bronchial contractions elicited by IHDA. Generally, wheezers had a worse airway status, consistent with the view that wheezing may be a sign of airway obstruction [6]. Wheezers also had a higher BMI than nonwheezers, but there was no difference in BMI between responders and nonresponders. Obesity per se may be a risk factor for airways obstruction and wheezing but not for hyperresponsiveness [33, 34]. Our results are comparable to previously published data in a population-based longitudinal study using histamine challenge in the sense that hyperresponsive subjects tended to develop respiratory symptoms more often than those with no proven BHR [6].

We observed significant deteriorations in baseline pulmonary function as assessed by IOS in responders, but not in nonresponders or healthy volunteers, and this divergence occurred after just a 3-year observation period. It is therefore concluded that responsiveness to IHDA in smokers may predict a decline in airway status over such a short period of time. It is not known if BHR to IHDA is associated with a worse prognosis in the long run, corresponding to previous findings in studies on BHR to methacholine challenge [5]. The IOS technique requires minimal patient cooperation, is particularly suitable for serial measurements such as those required during a bronchial challenge, and tends to be more sensitive than forced expirations in measuring discrete changes in airway function. Although such changes may be detected by IOS, the final long-term clinical relevance of the presence of BHR detected by IHDA is not known. Future studies on the long-term effect of smoking on BHR to IHDA and airway status may determine whether the choice of the cutoff limit of *F*_res_ after challenge indicates a true increased risk for the subject.
